# *Ornithodoros puertoricensis* (Ixodida: Argasidae) Associated With Domestic Fowl in Rural Dwellings From Córdoba Department, Caribbean Colombia

**DOI:** 10.3389/fvets.2021.704399

**Published:** 2021-06-23

**Authors:** Yesica López, Laura Natalia Robayo-Sánchez, Sebastián Muñoz-Leal, Ader Aleman, Esteban Arroyave, Alejandro Ramírez-Hernández, Jesús Alfredo Cortés-Vecino, Salim Mattar, Álvaro A. Faccini-Martínez

**Affiliations:** ^1^Instituto de Investigaciones Biológicas del Trópico, Universidad de Córdoba, Monteria, Colombia; ^2^Grupo Parasitología Veterinaria, Departamento de Salud Animal, Facultad de Medicina Veterinaria y de Zootecnia, Universidad Nacional de Colombia, Bogotá, Colombia; ^3^Departamento de Ciencia Animal, Facultad de Ciencias Veterinarias, Universidad de Concepción, Chillan, Chile; ^4^Department of Pathology, University of Texas Medical Branch, Galveston, TX, United States

**Keywords:** soft ticks, parasites, fowl nests, domiciliary infestation, *Ornithodoros*, Colombia

## Abstract

Ticks of genus *Ornithodoros* are nidicolous parasites associated with a wide array of vertebrates. In humans, their bites cause hypersensitivity reactions and are capable to transmit pathogens of health concern. In the department of Córdoba, Caribbean region of Colombia, the first report of an *Ornithodoros* soft tick was made in 1980 by Betancourt, who described the collection of *Ornithodoros talaje* in human dwellings. Nevertheless, current the records of *O. talaje* made in South America have been questioned and likely correspond to misidentifications with morphologically similar species. Between October and December of 2020, we visited rural areas of four localities from three municipalities within the department of Córdoba: Cuero Curtido and Severá (municipality of Cereté), El Espanto (municipality of Planeta Rica), and Arroyo Negro (municipality of San Carlos). Search for soft ticks was performed in 46 human domiciles and peridomiciliary areas. We searched in areas frequented by domestic animals, inspecting cracks in the walls and fowl nests. Infestation by soft ticks was found in 13% (6/46) of visited houses. Overall, 215 ticks were collected (26 larvae, 144 nymphs and 45 adults) from nests of domestic birds or in the adjacent walls. Larvae, nymphs and adults were morphologically identified as *Ornithodoros puertoricensis*. Molecular identification of ticks was confirmed by sequencing the tick mitochondrial 16S gene of adults, pools of nymphs and larvae. Pairwise comparisons showed a 99% of identity with *O. puertoricensis* from Panama. This study reports for the first time *O. puertoricensis* associated with domestic fowl in rural dwellings in Colombia, and expands the geographical distribution of this tick species toward the Córdoba department. Importantly, local people described exposure to tick bites while sleeping in infested houses; therefore, the transmission of soft tick-borne pathogens is now of concern in the region.

## Introduction

Ticks of the genus *Ornithodoros* are nidicolous arthropods that parasite a wide range of vertebrates, such as reptiles, birds and mammals, including humans (1). The saliva of *Ornithodoros* ticks can cause toxicosis (hypersensitivity and immunological response ranging from mild dermal lesions to systemic disease) (2) and transmit pathogenic agents, such as tick-borne relapsing fever (TBRF) group borreliae, to humans and domestic animals (3). Particularly in South America, *Ornithodoros brasiliensis, Ornithodoros fonsecai, Ornithodoros mimon, Ornithodoros rietcorreai, Ornithodoros rioplatensis, Ornithodoros rostratus*, and *Ornithodoros spheniscus* cause toxicosis (4–10). Moreover, human cases of TBRF were described during the first decade of the 20th century mainly in Colombia and Venezuela, and *Ornithodoros rudis* was involved as the vector (11–13). Interestingly, in the last two decades, studies have molecularly identified putative novel species of relapsing fever group borreliae in countries without reported cases, such as Chile and Brazil (14, 15).

In Colombia, while the study of TBRF vanished decades ago, 10 species of *Ornithodoros* are known to occur, namely *Ornithodoros azteci, Ornithodoros furcosus, Ornithodoros hasei, Ornithodoros marinkellei, Ornithodoros marmosae, Ornithodoros peropteryx, Ornithodoros puertoricensis, Ornithodoros rossi, Ornithodoros talaje*, and *Ornithodoros yumatensis* (16). Noteworthy, *O. rudis, O. furcosus*, and *O. puertoricensis* have been reported infesting human dwellings in the country (17–19). Adults of some Neotropical soft ticks are morphologically similar, and early reports of these three species was subject of confusion in Central and South America (16, 17). Therefore, old reports of *Ornithodoros* spp. made in Colombia need confirmation.

*Ornithodoros puertoricensis* was reported in Colombia for the first time in Ayacucho, in Cesar department, Caribbean region (20). Several years later, Betancourt (21) made the sole record of an *Ornithodoros* soft tick in the department of Córdoba (also in the Caribbean region). Specifically, the specimens were collected inside human dwellings at San Carlos municipality. At that time, the ticks were sent to the University of California and identified as *O. talaje* (21). Currently, the records of *O. talaje* made in South America have been questioned (16, 22) and likely correspond to misidentifications with morphologically similar species of the group. Therefore, the species reported by Betancourt (21) remains to be confirmed.

Fowl are common host for soft ticks of genus *Argas* (23) and there is evidence that *Ornithodoros* spp. ticks do parasitize domestic birds in Central America (20). Although 10 species of *Ornithodoros* occur in Colombia, fowl have never been implicated as hosts.

We carried out a prospective study in order to confirm the presence and identity of neglected *Ornithodoros* in rural dwellings from the department of Córdoba, including the San Carlos municipality. Our results demonstrate that soft ticks do occur in that region of Colombia and infest fowl nests.

## Materials and Methods

### Geographic Area and Sampling Sites

**Figure 1:**
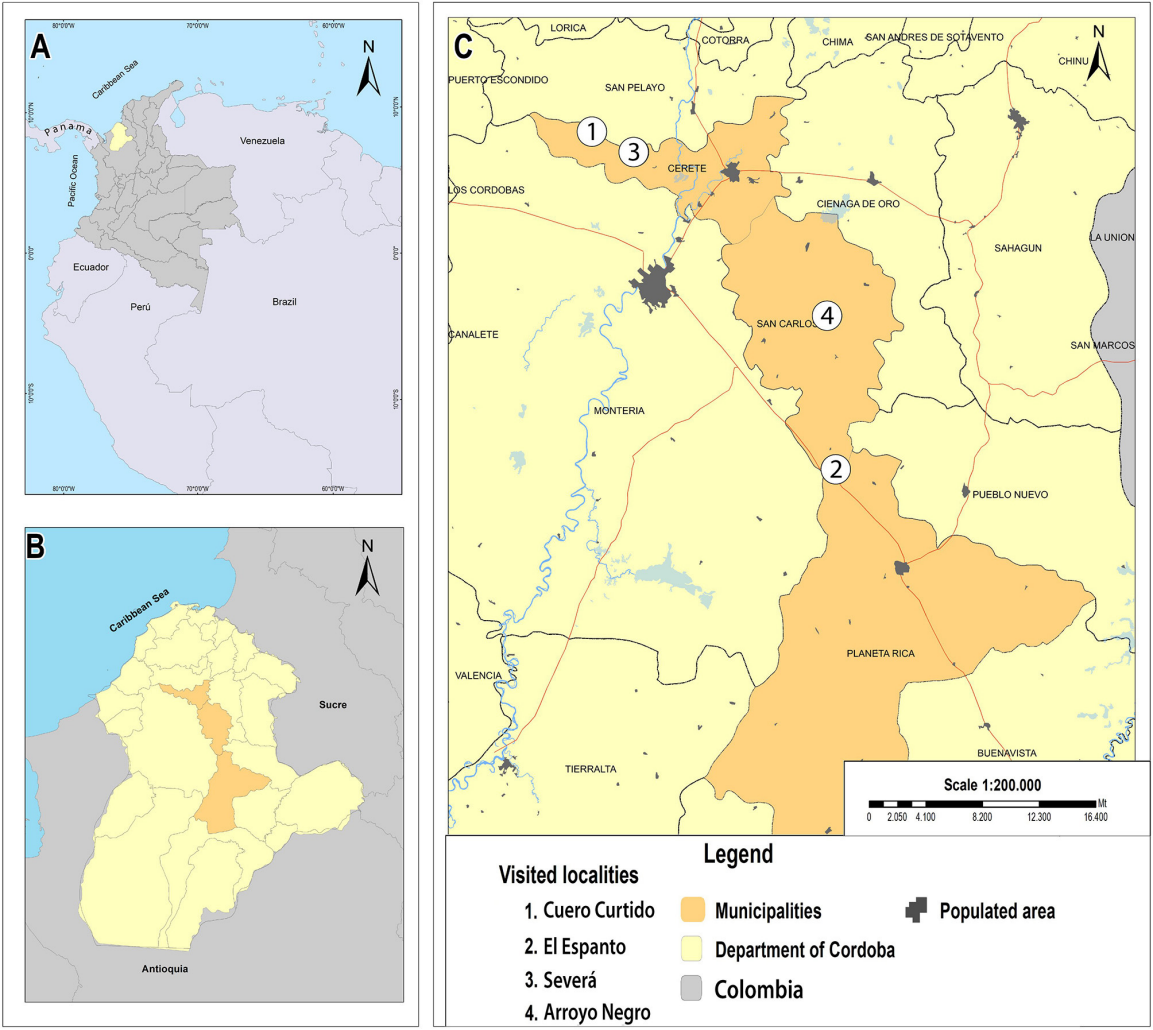
Map showing the collection locations. **(A)** Map of South America showing the location of Córdoba department within Colombia. **(B)** Map of Córdoba department showing the investigated municipalities. **(C)** Studied localities within the Cereté, San Carlos, and Planeta Rica municipalities.

Between October and December of 2020, we visited rural areas of four localities in three municipalities within the department of Córdoba, Colombia: Cuero Curtido (“locality 1”) and Severá (“locality 3”) (N 08°55'53” –W 75°57'31” and N 08° 90'77” –W 75°87'54”, respectively) in the municipality of Cereté; El Espanto (“locality 2”) (N 08° 31'46” –W 75°39'51”) in the municipality of Planeta Rica; and Arroyo Negro (“locality 4”) (N 08°42 '46“ –W 75°40'21”) in the municipality of San Carlos (Figure 1).

The three municipalities share similar environmental conditions (12–87 m of altitude, temperature between 25 and 28°C, and average relative humidity of 81%) (24–26). Municipality of Cereté has a population of 105,815 inhabitants (26), Planeta Rica of 64,205 inhabitants (24), and San Carlos of 23,532 inhabitants (25). The four visited localities were selected by convenience criteria, considering the easiness of access, and the researchers' safety regarding public order conditions in the area.

Searches for *Ornithodoros* ticks were performed in 46 human domiciles and peridomiciliary areas (eight houses in locality 1, three in locality 2, eight in locality 3, and twenty-seven in locality 4). The houses were constructed with wood, “bahareque” (clay or mud with sticks or canes), and “guadua” (woody bamboo) in their walls, reinforced with cardboard. All had dirt floors and palm leaves as roofs. The presence of domestic animals such as dogs, cats, pigs, chickens, turkeys, and ducks was common in all investigated houses, and the animals circulated freely inside the houses and in the peridomiciliary area. We examined domestic animals' resting areas, chicken coops, and nests of chickens, ducks and turkeys. Around these areas, cracks in the walls and nest debris were inspected. Inspections inside the inhabitants' rooms were not allowed for privacy reasons in most of the houses.

### Tick Collection and Identification

While larvae, nymphs, adults and exuviae were collected in 70% ethanol, eggs were maintained alive, transported to the laboratory, and kept in darkness inside an incubator (25°C, 80% relative humidity). Hatched larvae were mounted onto slides with Hoyer's medium and examined by light microscopy for morphological identification (27). A subset of adults was prepared for scanning electron microscopic examinations for greater definition of morphological characteristics. Larvae were morphologically identified following taxonomical keys (28). Morphological identification of adults and nymphs was done with original descriptions of *Ornithodoros* spp. (27, 29).

### Molecular and Phylogenetic Analyses

Morphological identification of ticks was complemented by molecular analysis. For this purpose, DNA extraction (QIAGEN DNeasy Blood & Tissue kit) was performed on individual adults, and larvae and nymphs of each locality were pooled (up to 10 larvae, up to 4 nymphs). Successful extractions were confirmed by PCR targeting the tick mitochondrial 16S gene for each sample with primers described elsewhere (30). Amplicons of expected size were Sanger-sequenced at the Molecular Genomics Core of the University of Texas Medical Branch (Galveston, TX). Obtained sequences were assembled with Geneious (31) and the consensuses compared with sequences available in GenBank using BLASTn (32).

An alignment with 33 sequences of Argasidae retrieved from GenBank was constructed in MAFFT (33). A phylogenetic analysis using the approximately maximum likelihood method was implemented in FastTree 2 (34), selecting the GTR model and five rates categories of sites. *Ornithodoros brasiliensis* (GU198363) and *Ornithodoros rostratus* (DQ295780) sequences rooted the tree.

## Results

### Tick Collection

**Figure 2:**
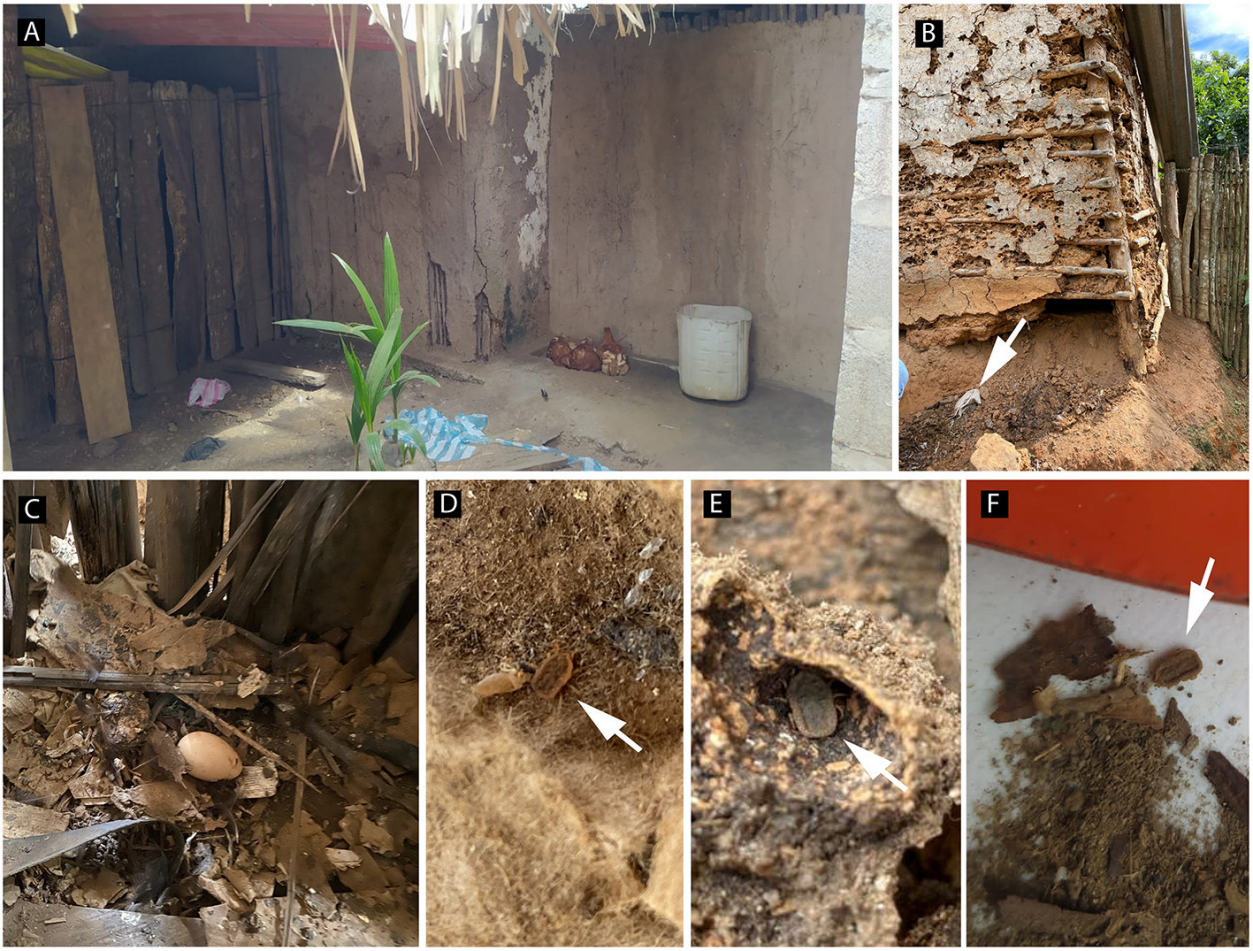
Dwellings where soft ticks were collected in the Córdoba department, Colombia. **(A)** Chickens lying adjacent to an external bahareque wall in locality 4 (Arroyo Negro); **(B)** Bahareque construction; note the crevices on the wall and the bird nest (arrow) in locality 2 (El Espanto); **(C)** chicken nest adjacent to the wall inside a dwelling in locality 1 (Cuero Curtido); **(D,F)** *Ornithodoros* ticks (arrow) collected in fowl nest debris in locality 1 (Cuero Curtido) and locality 3 (Severá), respectively; **(E)** *Ornithodoros* ticks (arrow) collected in between bahareque debris from a dwellings wall in locality 2 (El Espanto).

Infestation by *Ornithodoros* ticks was found in 12% (1/8) of the visited houses in locality 1, in 33% (1/3) of locality 2, in 25% (2/8) of locality 3, and 7% (2/27) of locality 4. Overall, 215 ticks were collected (26 larvae, 144 nymphs, 13 females, and 32 males) (Table 1). All were found in fowl nests and on adjacent walls. Briefly, 48 ticks were collected in locality 1, of which eight were adults (males) and 40 were nymphs, all collected in a hen's nest in the kitchen of a house with a dirt floor and cardboard-reinforced-wooden walls (Figures 2C,D). In locality 2 we found exuviae during inspection of the substrate (soil/sand) of a chicken nest adjacent to an external wall of a warehouse made of bahareque (Figure 2B). In this wall we collected 32 ticks (four females, two males, 26 larvae and a group of eggs) (Figure 2E). Interestingly, the owners of the warehouse recognized the ticks, named them as “Pitos,” and also referred to bites of these arthropods while sleeping. In locality 3 a total of 121 ticks [27 adults (six females, 21 males) and 94 nymphs] were collected in chicken nests made of dried-banana-leaves, found in a warehouse and a kitchen, respectively, of two houses with guadua walls, dirt floor, and palm roof (Figure 2F). In locality 4, 14 ticks [four adults (three females and one male) and 10 nymphs] were collected in bahareque walls adjacent to chicken nests, in a warehouse and a room of two houses, respectively (Figure 2A).

**Table 1:** *Ornithodoros* spp. collected in rural dwellings from Córdoba department, Colombia.

**Municipality**	**Locality**	**Collection area**	**Total of collected specimens**	**Number of ticks submitted to DNA extraction**	**Individual/pool DNA extraction**
Cereté	1 (Cuero Curtido)	Hen's nest	8♂, 40N	8♂	Adults individually
Cereté	3 (Severá)	Chicken nests	21♂, 6♀, 94N	3♂, 4♀, 10N	Adults individually/3 pools (one of 2N; two of 4N)
San Carlos	4 (Arroyo Negro)	Bahareque walls	1♂, 3♀, 10N	1♂, 3♀, 10N	Adults individually/6 pools (three of 1N; two of 2N, one of 3N)
Planeta Rica	2 (El Espanto)	Bahareque wall	2♂, 4♀, 26L	2♂, 3♀, 10L	Adults individually/1 pool of 10L
Total			215	54	34

*♂, Males; ♀, Females; N, nymphs; L, Larvae*.

Remarkably, domiciles where we found ticks were comparatively rudimental and precarious; had poor hygiene, and sanitary conditions; dirt in the most of their floors, and no record of recent pest chemical control.

### Morphological and Genetic Identification of Specimens

**Figure 3:**
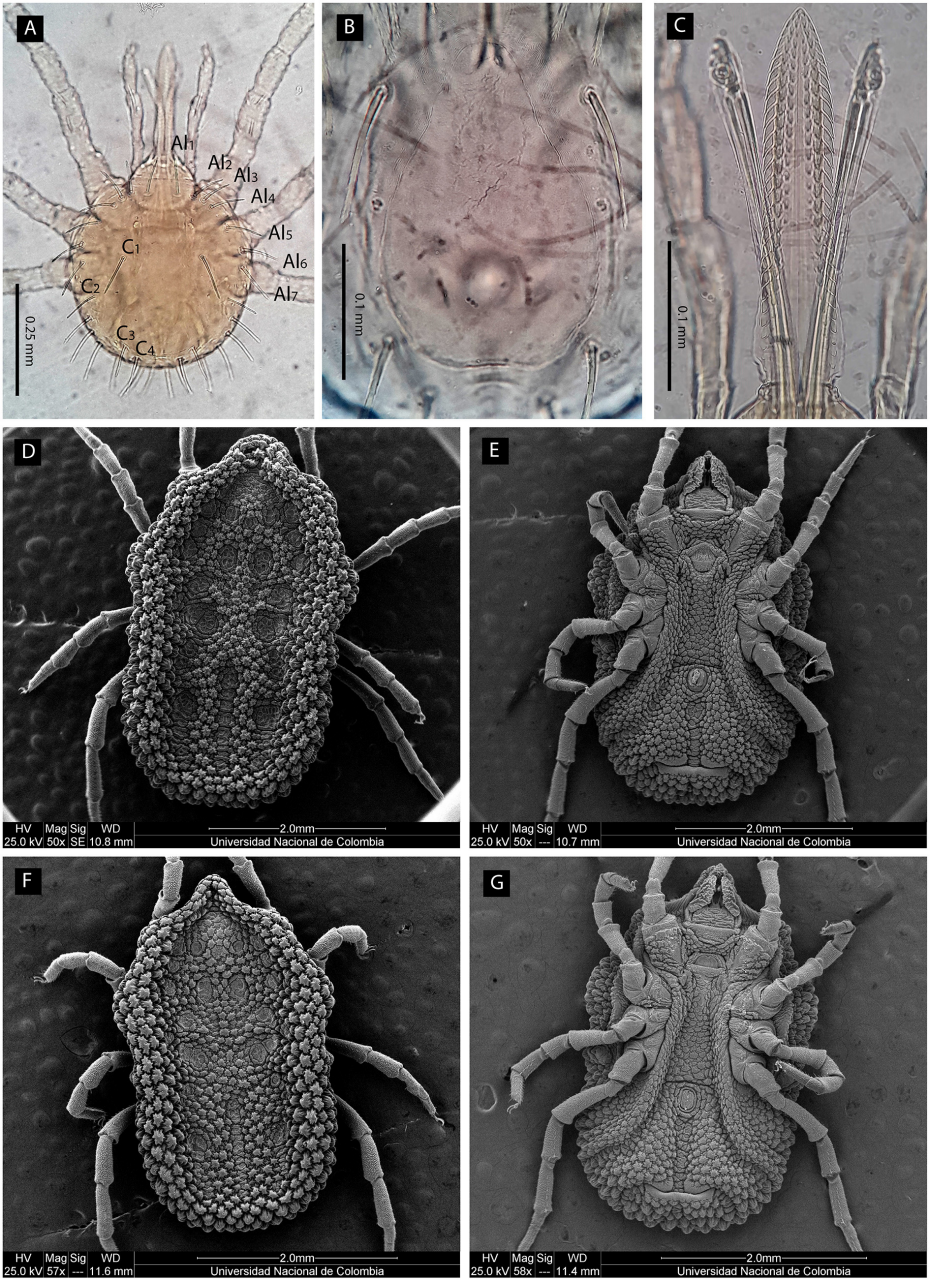
Optical and scanning electron microscopic micrographs of *O. puertoricensis* collected in Córdoba department, Colombia. Larva: **(A)** dorsal view, **(B)** dorsal plate, and **(C)** hypostome. Female: **(D)** dorsal and **(E)** ventral views. Male: **(F)** dorsal and **(G)** ventral views. Al, anterolateral setae; C, central setae.

Based on the examination of 10 slide mounted specimens, larvae were identified morphologically as *O. puertoricensis* because of the following combination of traits: dorsal plate pyriform; 17–18 pairs of dorsal setae (seven anterolateral, 6–7 posterolateral and four central); hypostome pointed, with dentition formula 3/3 in middle length (Figures 3A–C). Nymphs and adults were identified morphologically as the same species by the combination of the following characters: presence of cheeks, conical mammillae, anteromedian disk present, posteromedian file of disks merging with median disk (Figures 3D–G). Examined larvae and adults were deposited in the “Colección Parasitológica Veterinaria Julio Mario Rodríguez Peña” at the Universidad Nacional de Colombia (UNAL: CPV-UN: 2021ACAR001–003). Genetic identification of ticks was performed individually for 14 males, 10 females, nine pools of nymphs and one pool of larvae collected in the four localities (Table 1). Two haplotypes with two polymorphisms consisting of adenine-guanine transitions were retrieved (99.5% of identity between them). Both sequences were 99.2–99.7% identical to *O. puertoricensis* from Panama available in GenBank (KX685710) (35). Haplotype I was found in 28 pools of ticks (14 males, nine females and five pools of nymphs) from the four localities, while haplotype II was only found in six pools of ticks (one female, four pools of nymphs and one pool of larvae) from two of the localities (El Espanto and Arroyo Negro). Tick mitochondrial 16S rDNA sequences of *O. puertoricensis* generated in this study were deposited in GenBank under accession numbers MZ005589 and MZ005590.

**Figure 4:**
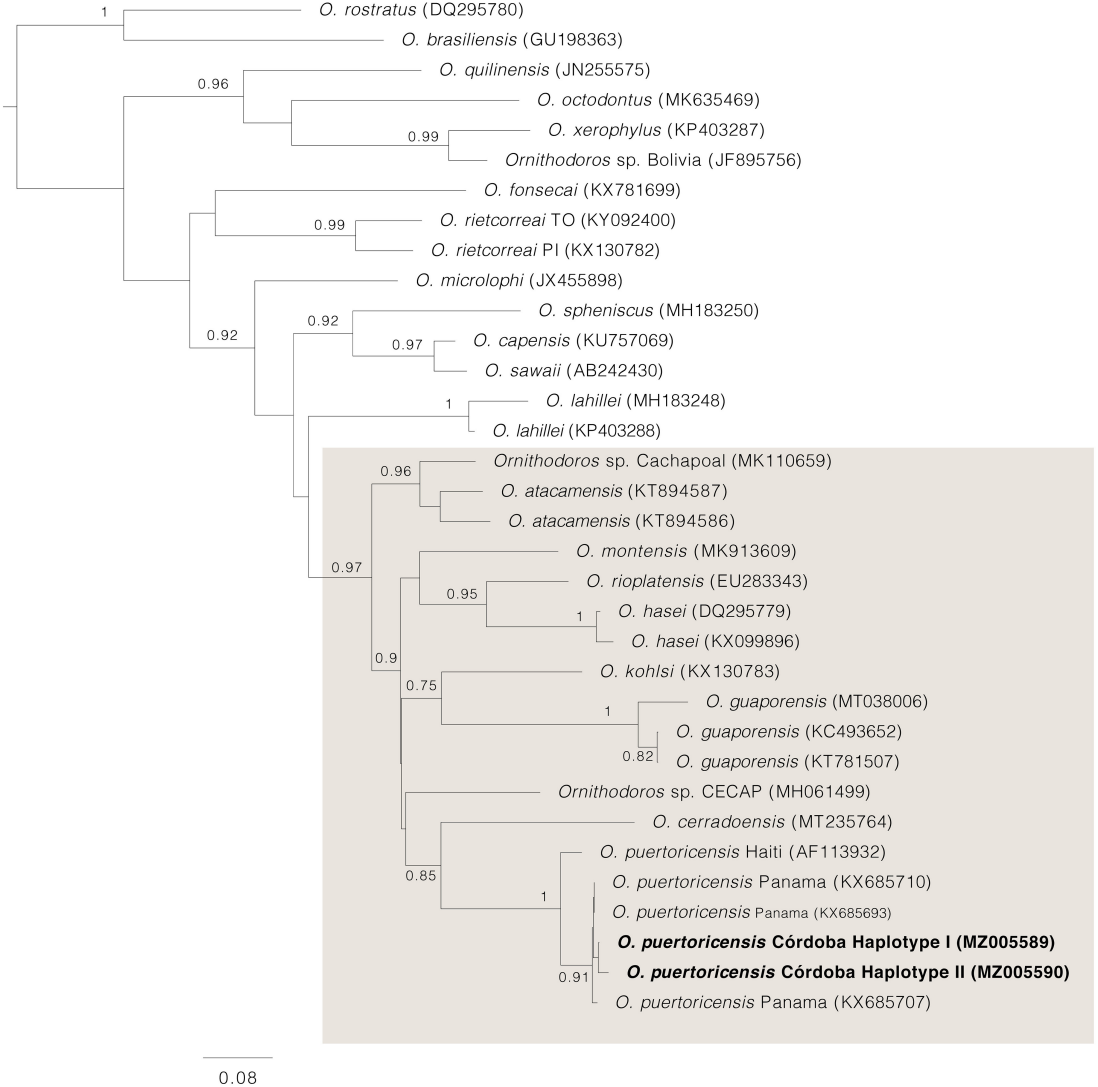
Approximately maximum likelihood phylogenetic tree for a subset of *Ornithodoros* spp. Support values >0.75 are shown above or below main branches. The position of *O. puertoricensis* collected at Córdoba department is highlighted in bold. The *O. talaje* group is boxed in gray.

The phylogenetic analysis determined that *O. puertoricensis* from the Córdoba department in Colombia are closely related to a homologous species from Panama. A previous sequence of *O. puertoricensis* from Haiti (AF113932) branches basally to the Colombian and Panamanian ticks. Collectively, the sequences of *O. puertoricensis* cluster as a sister group to *Ornithodoros cerradoensis* (Figure 4). This topology is consistent with previous phylogenies including the *O. talaje* group (27).

## Discussion

This study reports for the first time *O. puertoricensis* infesting domestic fowl nests in rural dwellings in Colombia, and expands the geographical range of this tick species to the Córdoba department. However, *O. puertoricensis* is no stranger, since it has been reported in the northwestern region of the country. The first record of *O. puertoricensis* in Colombia was published by Fairchild in 1966, referring to adult specimens collected in the Ayacucho municipality, in Cesar department (20). Later, in 2009, Paternina et al. described the finding of larvae infesting dogs in the Sucre department (36), and recently, larvae and females were described infesting synanthropic rodents (i.e., *Mus, Rattus*) and human dwellings in Urabá region, in the Antioquia department (19, 37, 38). In addition, Butler and Gibbs (39) stated that *O. puertoricensis* occurs in the Colombian pacific region as well.

*Ornithodoros puertoricensis* was described based on larvae collected on rats (*Rattus* spp.) in Puerto Rico (40). Moreover, amphibians, reptiles and mammals host larvae of *O. puertoricensis* in Jamaica (*Herpestes javanicus, Nectomys* sp., and *Proechimys* sp.), Panama (*Rattus* sp., *Sylvilagus brasiliensis, Eyra barbara, Dasyprocta punctata, Felis silvestris catus, Rhinella marina, Varanus dumerilii, Python regius, Python bivittatus*, and *Homo sapiens*), Trinidad (*Proechimys trinitatus* and *Nectomys squamipes*), Nicaragua (*Dasyprocta punctata* and *Didelphis marsupialis*), Venezuela (*Proechimys guyannensis, Proechimys semispinosus, Dasyprocta fuliginosa, Sigmodon alstoni, Zygodontomys brevicauda, Sylvilagus floridanus, Tamandua tetradactyla, Conepatus semistriatus, Monodelphis brevicaudata, Marmosa robinsoni, Artibeus lituratus, Iguana* sp., and unidentified lizard), and Puerto Rico (*Felis silvestris catus*) (20, 35, 41–46). In particular, the sole record of *O. puertoricensis* associated with birds comes from Mexico, where larvae were collected on *Speotyto cunicularia* (29). Meanwhile, Dunn in 1931 collected larvae and adults of *O. talaje* on chickens and chicken coops in the Panama City market (20). Nevertheless, records of *O. talaje* in Panama prior to 1947 are questionable because of morphological confusion with *O. puertoricensis* (20, 40). Dunn's descriptions are similar to our findings in that they suggest that *Ornithodoros* soft ticks do associate with domestic fowl. Even though in the present study we did not collect larvae feeding on animals, it is likely fowl could maintain the biological cycle of *O. puertoricensis* in the visited localities, since eggs, larvae, nymphs and adults were taken from their nests. Thus, our results suggest that the previous report of *O. talaje* made by Betancourt (21) at the same locations probably corresponds to *O. puertoricensis*.

*Ornithodoros puertoricensis* do infest human dwellings in Panama and Colombia (19, 35) and mild toxicosis was reported after their bites (35). Inside houses, *O. puertoricensis* seems to parasitize humans during the night (35). Indeed, during our investigations, the inhabitants of infested houses recognized the ticks and reported nocturnal parasitism as well. Interestingly, the common name for *O. puertoricensis* in the region (i.e., “Pito”), is the same one assigned to triatomine bugs, vectors of Chagas disease in Colombia (47). To acknowledge this coincidence in common names is valuable from an epidemiological point of view and in further inquiries looking for soft ticks infesting domiciliary environments in the region. Finally, evidence that *O. puertoricensis* transmits human pathogens is currently lacking. However, the role as a vector should not be ruled out, since other *Ornithodoros* ticks are reservoirs of TBRF spirochetes of human health concern.

## Data Availability Statement

The datasets presented in this study can be found in online repositories. The names of the repository/repositories and accession number(s) can be found at: https://www.ncbi.nlm.nih.gov/genbank/, MZ005589; https://www.ncbi.nlm.nih.gov/genbank/, MZ005590.

## Author Contributions

YL, SM-L, SM, and ÁF-M designed the initial study. YL, AA, and ÁF-M carried out the field work. YL, SM-L, LR-S, AR-H, and JC-V performed the tick identification and tick processing. YL, SM-L, EA, and ÁF-M implemented the molecular analysis. YL, SM-L, and ÁF-M wrote the first draft of the manuscript. All authors contributed to data interpretation and revisions.

## Conflict of Interest

The authors declare that the research was conducted in the absence of any commercial or financial relationships that could be construed as a potential conflict of interest.
